# Engaging people living with dementia and care partners in the design, decision‐making, and activities of a national knowledge hub to support community‐based dementia initiatives: The Canadian Dementia Learning and Resource Network (CDLRN)

**DOI:** 10.1002/alz70858_106985

**Published:** 2025-12-25

**Authors:** Laura E. Middleton, Danielle Krisman, Christine Pellegrino, Dana Zummach, Carrie A McAiney

**Affiliations:** ^1^ Schlegel‐UW Research Institute for Aging, Waterloo, ON, Canada; ^2^ University of Waterloo, Waterloo, ON, Canada

## Abstract

Canada's National Dementia Strategy specifies improving the quality of life of people living with dementia and family/friend care partners as a key objective. To this end, the Public Health Agency of Canada established the Dementia Community Investment (DCI), a funding steam to support projects that design, implement, and evaluate community‐based initiatives aimed at enhancing quality of life for people impacted by dementia. To support the capacity and impact of DCI‐funded projects, the Public Health Agency of Canada also funded the Canadian Dementia Learning and Resource Network (CDLRN) starting in 2020. CDLRN provides education and capacity‐building to DCI‐funded projects and shares their successes to amplify impact to communities, and particularly to people living with dementia. The objective of this presentation is to describe the engagement of people living with dementia and care partners in the design, decision‐making, and activities of CDLRN. Since the conception of CDLRN, engagement of people living with dementia and care partners has been a key priority. We are guided by the principles and enablers of Authentic Partnership, which emphasize a genuine respect for others, valuing diverse perspectives, a focus on open and responsive processes, and regular reflection and dialogue. CDLRN's structures and processes were co‐designed by network members, with people living with dementia engaged as project representatives and CDLRN advisors. This engagement shaped the name of the network, the network website, and processes for communications and events. The CDLRN Community Advisory Committee provides strategic guidance for CDLRN, with several people living with dementia as members, ensuring that their voices and priorities guide decision‐making. People living with dementia also participate in and present at nearly all CDLRN events and activities. For example, the CDLRN annual forum starts with a panel of people living with dementia to guide our learning and thinking. People living with dementia are also members of evaluation co‐design teams and working groups. Engaging people living with dementia and care partners in the development of CDLRN events and activities has resulted in a knowledge hub that is flexible and responsive to the evolving needs of the DCI‐funded projects.

